# A Comparison of Analgesic and Recovery Profiles of Ketamine, Lignocaine, and Dexmedetomidine (KeLiDex) Versus Fentanyl-Based Anesthesia in Laparoscopic Nephrectomies: A Randomized, Single-Blind, Pilot Study

**DOI:** 10.7759/cureus.63380

**Published:** 2024-06-28

**Authors:** T. C. Arun, Habib Md R Karim, Subrata K Singha, Deepak K Biswal

**Affiliations:** 1 Anaesthesiology, Critical Care, and Pain Medicine, All India Institute of Medical Sciences, Raipur, Raipur, IND; 2 Anaesthesiology, Critical Care, and Pain Medicine, All India Institute of Medical Sciences, Guwahati, Guwahati, IND; 3 Urology, All India Institute of Medical Sciences, Raipur, Raipur, IND

**Keywords:** rescue analgesia, post-operative recovery, anesthesia recovery period, opioid free analgesics, opioid free anesthesia, kelidex

## Abstract

Background: In the search for opioid-free anesthesia, notable numbers of drugs, singly or in combinations, have been tested with variable results. However, most of the drugs used are not as strong as opioids. Even if some non-opioid drugs are potent enough, they cause significant untoward effects, necessitating the use of lower effective dosages of multiple drugs as a substitute. The present pilot study evaluated low-dose combinations of ketamine, lignocaine, and dexmedetomidine (KeLiDex) against fentanyl-based anesthesia for analgesia and recovery profiles in laparoscopic nephrectomies.

Methods: Twenty patients (10 in each group) randomly received KeLiDex or fentanyl infusion as an analgesic component for balanced general anesthesia. Entire patients also received paracetamol and quadratus lumborum block-2. Anesthesia depth, neuromuscular blockade, and reversal were standardized. Intraoperative hemodynamic variation, time to extubation after reversal (T-tEAR) administration, postanesthesia care unit (PACU) discharge readiness assessed using modified Aldrete score, sedations using Richmond Agitation Sedation Scale, postoperative pain, and rescue analgesia consumptions were compared using different validated scales. P-value <0.05 was considered significant.

Results: The KeLiDex group had a significantly lower heart rate (HR) between 45-90 minutes and at the time of reversal. Mean arterial pressure (MAP) (mean ± standard deviation (SD)) differed significantly at only a 60-minute interval (KeLiDex group 80.90 ± 9.50 versus fentanyl group 92.60 ± 16.13 mmHg, p-value 0.041). The Friedman test for change in HR and MAP over time within each group was also insignificant. The mean ± SD of T-tEAR was 6.37 ± 2.13 in KeLiDex, and 8.18 ± 2.92 minutes in the fentanyl group, p-value 0.27. Sedation scores, Modified Alderette scores, pain scores, and rescue analgesic requirements were also comparable.

Conclusion: KeLiDex could effectively control hemodynamics and pain both at rest and in movements in line with fentanyl-based anesthesia for laparoscopic nephrectomies. Further, recovery from the anesthesia, sedation, and PACU discharge readiness were similar.

## Introduction

Global data indicates that kidney malignancies constitute about 2.2% of all new cancer cases and 1.8% of all new deaths [1]. Notably, most cases present within 50-60 years of age, making it socially and economically impactful [2]. Nephrectomy is a curative treatment modality for kidney cancer patients. Yet, there are other indications for nephrectomies and non-functioning kidneys, mainly secondary to obstructive uropathy, which constitutes another central entity that requires nephrectomies [3,4]. Laparoscopic nephrectomies have vast benefits compared to open surgeries - having less postoperative pain requiring less analgesia, early recovery resulting in lower hospital stays, and resumption of regular activity [5]. Although laparoscopic nephrectomies have lower pain compared to open procedures, they still require potent opioids in the form of an infusion set or patient-control analgesia pump, along with regular drugs like paracetamol and tramadol.

While opioids are time-tested potent analgesics, they have serious side effects, including potentially life-threatening respiratory depression, opioid-induced hyperalgesia, and addiction. Furthermore, context-sensitive half-time for most opioids is a crucial aspect that needs consideration while administering for a longer infusion duration. These factors have driven the perioperative practice from opioid-based anesthesia to opioid-free anesthesia (OFA). Multiple analgesic agents and multiple techniques, including regional and neuraxial, with different mechanisms of action, have proven their efficacy as opioid-sparing and alternative agents in perioperative care; Ketamine, lignocaine, and dexmedetomidine (KeLiDex) are also a few of them [6]. While there has been significant research on OFA, most of the opioid-sparing drugs alone are not potent enough to substitute opioids in the perioperative period. Therefore, perioperative physicians are frequently choosing multiple agents to achieve the goal.

Furthermore, despite being an effective model, epidural analgesia is sometimes not preferred in cases with renal vein thrombosis and inferior vena cava involvement due to the requirement of anticoagulation [7]. Multimodal use of analgesics has been the recent standard where the technique can also reduce the use of opioids and the further complications due to opioids. Transverse abdominal plane blocks, erector spinae blocks, quadratus lumborum blocks, paravertebral blocks, etc. are considered a safe yet effective alternative to epidural [8]. Considering the mentioned facts, the present study aimed to examine the analgesic and recovery profile of KeLiDex combination and compare them with fentanyl-based anesthesia for laparoscopic nephrectomies in a background of multimodal analgesia practice. The study's primary aim was to compare the hemodynamic variation as a surrogate marker of adequate analgesia under standardized and controlled balanced anesthesia. We hypothesized that KelLiDex might be better than fentanyl in maintaining hemodynamics and preventing variation from baseline.

## Materials and methods

Study design and settings

The present study was a randomized, single-blind, parallel-arm, single-center pilot study conducted in an academic institute in India. The study protocol was evaluated by the Postgraduate Thesis Review Committee and examined and approved by the Institute Ethical Committee. The study was initiated after registering with the Clinical Trial Registry India and informed and written consent was obtained from each participant. The study was conducted in the operating theatres and post-anesthesia care unit (PACU) with a ward follow-up. The participants were recruited from March 2022 to March 2023.

Study population

Patients undergoing general anesthesia (GA) with endotracheal intubation for transperitoneal laparoscopic nephrectomies were eligible for the study recruitment. Other inclusion criteria were males or females gender, adults up to 70 years, with body mass index (BMI) between 18.5 and 29.99 kg/m^2^ and falling with American Society of Anesthesiologists physical status (ASA-PS) class I-III. The exclusion criteria were patient's refusal, patients with a diagnosis of shock or sepsis, patients with significant and severe organ failure requiring support (respiratory, liver, cardiac, renal), patients with uncontrolled hypertension (>180/100 mmHg), patients with suspected or known autonomic neuropathy, special population like jailed personals, protected tribes, etc., preoperative bradycardia or atrioventricular (AV) block intraoperative circumstances like vasopressor or vasodilation infusion if required or requiring hypotensive anesthesia, and airway management requiring unconventional technique and modification of induction and oxygenation, or patient is not extubated till six-hour postoperative period due to non-intervention related cause were also excluded.

Randomization, allocation, and concealment

Eligible and consented participants were randomized using the block randomization technique. Twenty-four random codes, comprising six blocks of four codes per block, were generated using the sealed envelope^TM^ (www.sealedenvelope.com; Sealed Envelope, London, UK), an online randomization service. After identifying prospective participants and confirming their informed consent, they were allocated into two groups. Group A received KeLiDex infusion, while Group B received fentanyl infusion as an intervention drug. Block allocation followed a sequential order, and concealment was maintained using the code number of treatments. The code number and treatment were available only to the principal investigator. However, due to the inherent nature of the technique, only the participants were blinded to the intervention; neither the performer nor the evaluator were blinded.

Sample size

The study was planned with a confidence of 95%, which gave a sample of a minimum of three and a maximum of eight patients for a hypothesized probability of getting a difference between 33% (in one-third of patients) and 67% (two-thirds of cases); the maximum number, i.e., eight, was chosen. The sample size was calculated using http://www.crutzen.net/n.htm based on the Viechtbauer et al.'s formula [9]. A margin of 20% was added to the sample size for drop-out or exclusion, and a design effect of 1.0 (considering randomized design) reached a final sample of 10 patients per group, a total of 20 participants.

Techniques

All patients received the anti-anxiety drug tablet lorazepam 2mg the night before surgery and tab ranitidine 150 mg the night before and in the morning. No patient received any pre-medications having analgesic activity. GA induction was standardized. Invasive arterial blood pressure was measured on the non-dominant hand using radial artery cannulation during the intraoperative and immediate postoperative periods. Both groups received intravenous lignocaine 2% 2 mL before propofol injection to blunt the tracheal intubation response as well as propofol injection-related pain. Patients in Group A received a loading dose of ketamine 0.5mg/kg in 10mL volume to simulate the fentanyl loading dose of 2 mcg/kg for Group B.

Further, ketamine 0.15mg/kg/h + dexmedetomidine 0.2 mcg/kg/h and lignocaine 0.25 mg/kg/h were given as an infusion in Group A. The mixture was examined for visible sediment under the microscope. However, despite not showing any sediment, we planned to infuse the drugs separately. The drug doses are kept lower considering the polypharmacy in nature and are based on the current literature. All syringes were marked as study drugs and coded as per randomization, whose number code was not available to the pain evaluator for maintaining blinding as far as possible, especially in the postoperative period. Group B received fentanyl 2 mcg/kg in 10 mL volume as a bolus followed by 0.5 mcg/kg/h infusion. Other GA induction drugs like propofol were used as per titrated to effect, and vecuronium (loading dose 0.1 mg/kg) to facilitate endotracheal intubation (ETI), and maintenance dose (1 mg bolus) was guided by neuromuscular blockade monitoring (NMT) and were the same for both groups. All the patients in both groups received quadratus lumborum block. The safe dose calculation for local anesthetics in the quadratus lumborum block-2 (QLB-2) was done after deducting the cumulative lignocaine infusion over the last four hours (two t1/2 of lignocaine). The drug used for QLB-2 was 0.25% bupivacaine. The block was performed in the lateral decubitus position, the same as the operating position. The linear probe was kept in the midaxillary line axial plane and moved posteriorly to find a suitable image and trajectory. A volume of 15 mL was used in both groups. All QLB-2 blocks were performed using the ultrasound-guided technique, and a Sonosite (Fujifilm Sonosite, Inc., Washington, United States) ultrasound machine was used to perform the blocks.

Both groups received low-flow anesthesia (fresh gas flow 600-800 mL/min) with a potent inhalational agent using nitrous oxide (N_2_O) + oxygen (O_2_) as vehicle gas titrated to age-adjusted minimum alveolar concentration (MACage) of 1.1 ± 0.1. Age monitoring was automatic from the workstation monitoring after entering the patient's demographics in the monitoring system [10]. All patients received intraoperative paracetamol. Inspiratory N_2_O and O_2_ titration was per continuous agent monitoring data, and a fraction of inspired N_2_O (FiN_2_O) was maintained between 55% and 65% during maintenance. All GA was induced and maintained using a Dräger Primus anesthesia workstation, vaporizer, and NMT monitoring (Drägerwerk AG & Co. KGaA, Germany).

The intervention drugs (KeLiDex and fentanyl) were stopped immediately after the nephrectomy specimen was removed. The QLB-2 blocks were performed after the incision closure for the nephrectomy procedure and before the GA reversal. The neuromuscular blockade reversal was done using the injection of neostigmine (50 mcg/kg, rounded to the nearest 0.5 mg) and glycopyrrolate (10 mcg/kg, rounded to the nearest 0.1 mg), administered at 10-20% of the train-of-four (TOF) ratio. All patients received injections of ondansetron 4 mg at the time of reversal. The GA and blocks were performed mostly by the same team.

Outcome and data collection

Over and above the patient's demographic and clinical data, data related to the objectives were collected in predefined case record form. As objective analgesia monitoring under anesthesia is currently not widely available in most of the world, intraoperative hemodynamic variation (tachycardia, increase in blood pressure, etc.) under adequate anesthetic depth is considered a surrogate of proper analgesia. The time to extubation after reversal (T-tEAR) was calculated from the time of reversal administration to the trachea's extubation. The reversal was administered when the TOF ratio was >20%. The extubation was done when the TOF ratio was >90%. The time required to reach the modified Alderete score >9/10 was noted. The pain score was measured using a numerical rating scale (NRS). Postoperative nausea and vomiting (PONV) were scored on a Likert scale of 0-4 (5 score classes). Pain assessment was done using an 11-point (0-10) NRS scoring at 10 minutes, 30 minutes at the PACU, and then at 6 hours, 12 hours, 24 hours, 36 hours, and 48 hours. Ward assessment values within one hour pre- or post-scheduled time were acceptable. Vitals and Richmond Agitation Sedation Scale (RASS) data were also noted at this time point. The time to first rescue analgesic was measured as the time from the extubation to the need for the first dose of analgesia as indicated by the patient's demand (pain complaint) or NRS score of more than or equal to 4/10. The first-line rescue analgesia was an injection of paracetamol 20 mg/kg, rounded to the nearest 50 mg (for example, for a 47 kg patient, the dose will be 47x20=940 mg, which will be rounded to the nearest 50mg division, i.e., 950 mg), maximum of 1 gm. All such patients were then kept on injection of paracetamol thrice daily for the first post-operative day; then, it was converted to 650 mg oral. Second-line rescue analgesia was the injection of tramadol 100 mg and escalated to intravenous nalbuphine 2 mg bolus if the pain control was still inadequate.

Data management and statistical analysis

A master chart was prepared in Microsoft Excel (Microsoft Corporation, Redmond, United States). Data was analyzed using the Statistical Package for Social Science (SPSS) version 23 (IBM SPSS Software, IBM Corporation, Chicago, United States). Data distribution was tested using the k-test, and the statistical test was based on the data distribution. Qualitative data was presented using numbers and percentage scales and compared using Fisher's exact test. Continuous parametric data was analyzed using an unpaired t-test. The Wilcoxon-Mann-Whitney test was used for non-parametric data that were not normally distributed. Friedman test was used to test the variation over time within the group. Contingency table analysis was done using the Chi-square test, and if any of the values were less than 5, Fisher's exact test was used. A p-value of <0.05 was considered significant.

## Results

A total of 23 patients were randomized, three of whom were converted into open nephrectomy cases (one from the KeLiDex group and two from the fentanyl group). Twelve (60%) were males, and eight (40%) were females. Only five (25%) cases were non-functioning kidneys, and all had normal creatinine and blood urea levels, categorized as ASA-PS status I. The remaining 15 (75%) were ASA-PS II and III. The baseline clinicodemographic variables were similar among the groups.

Entire patients received the intervention and other drugs as per protocol. The mean heart rate (HR) for both groups increased slightly from baseline at preoperative and laryngoscopy; however, the differences between the groups were insignificant. On the other hand, Group A (KeLiDex) had a significantly lower HR from 45 minutes to 90 minutes of anesthesia. Similar results were also noted at the time of reversal when the KeLiDex showed a lower increase in the HR and significantly lower absolute HR than Group B, i.e., fentanyl-based anesthesia. The change in HR over time within each group tested by the Friedman test did not show statistical significance. The mean ± standard deviation (SD) values over time and their comparison are shown in Table 1.

**Table 1:** Comparison of heart rates presented as mean ± standard deviation and their changes over time among the two groups at each point; p-value <0.05 was considered significant. ^1 ^P-value for comparison of the two groups at each time point (Wilcoxon-Mann-Whitney Test). ^2 ^P-value for change in heart rate over time within each group (Friedman Test). KeLiDex: ketamine, lignocaine, and dexmedetomidine; LETI: laryngoscopy and endotracheal intubation; T1 represents the immediate post-intubation time point; T2 represents the extubation time point.

Time Points	Group A (KeLiDex)	Group B (Fentanyl)	P-value^1^
Baseline	87.2 ± 7.69	91.6 ± 11.69	0.495
Preoperative	91.50 ± 11.9	95.8 ± 16.99	0.427
5 Minutes before LETI	96.0 ± 12.55	107.0 ± 20.61	0.127
Immediate Post-intubation (T1)	96.1 ± 12.49	110.0 ± 16.83	0.058
5 Minutes of T1	95.6 ± 13.57	107.7 ± 19.62	0.198
10 Minutes of T1	92.4 ± 11.75	103.1 ± 22.65	0.212
Incision	91.3 ± 8.59	100.7 ± 21.18	0.272
15 Minutes of T1	83.0 ± 10.89	95.2 ± 16.84	0.130
30 Minutes of T1	83.6 ± 7.01	95.0 ± 10.45	0.053
45 Minutes of T1	81.5 ± 11.16	96.5 ± 12.59	0.017
60 Minutes of T1	81.1 ± 12.38	96.3 ± 14.0	0.012
75 Minutes of T1	78.0 ± 8.82	96.9 ± 12.60	0.004
90 Minutes of T1	79.3 ± 6.91	95.67 ± 10.79	0.002
120 Minutes of T1	80.4 ± 13.83	94.44 ± 12.5	0.054
150 Minutes of T1	78.0 ± 10.86	89.0 ± 11.92	0.133
180 Minutes of T1	78.0 ± 6.48	87.2 ± 15.35	0.387
210 Minutes of T1	82.5 ± 14.85	85.33 ± 16.62	1.000
240 Minutes of T1	79.5 ± 12.02	89.67 ± 13.58	0.554
Reversal	78.7 ± 12.98	95.1 ± 13.16	0.013
Extubation (T2)	85.4 ± 9.62	94.9 ± 11.19	0.064
1 Minutes of T2	83.80 ± 8.46	90.1 ± 15.20	0.226
5 Minutes of T2	81.6 ± 9.48	90.0 ± 17.01	0.103
10 Minutes of T2	80.30 ± 8.60	86.80 ± 14.94	0.137
20 Minutes of T2	78.7 ± 7.62)	86.9 ± 13.47	0.088
30 Minutes of T2	77.3 ± 8.69	89.9 ± 16.56	0.075
40 Minutes of T2	76.9 ± 8.49	86.5 ± 14.46	0.103
50 Minutes of T2	75.3 ± 9.18	84.4 ± 15.12	0.082
60 Minutes of T2	75.9 ± 7.34	83.3 ± 15.13	0.149
P-value^2^	0.476	0.757	

**Figure 1:**
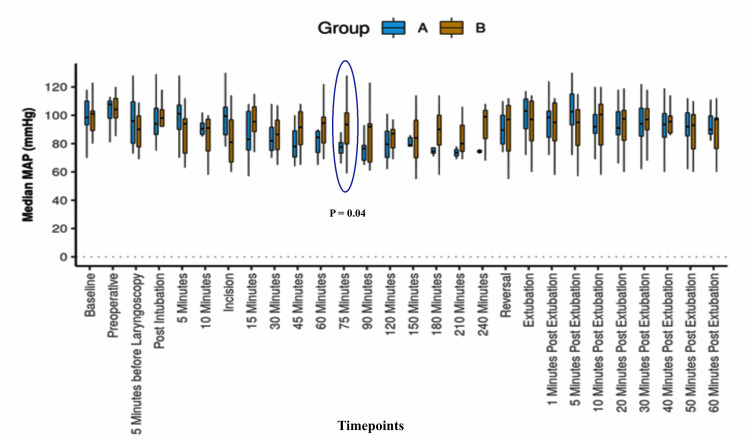
Box and whisker plot showing median, quartile, and range of MAP and comparison between groups across different time points; p-value <0.05 was considered significant. Group A: KeLiDex; Group B: fentanyl; MAP: mean arterial pressure; KeLiDex: ketamine, lignocaine, and dexmedetomidine

The mean ± SD and median of mean arterial pressure (MAP) between the groups differed significantly at only a 60-minute (Figure 1).

In both groups, the intragroup changes in mean MAP for different time points from the baseline were statistically indifferent. However, the intergroup changes for the 75-minute time point from the baseline were significantly different (Table 2).

**Table 2:** Comparison of absolute changes of the mean (standard deviation) differences in mean arterial pressure from baseline to follow-up time points; p-value <0.05 was considered significant. * P-value of change within the group. ^# ^P-value for comparison of the two groups in terms of differences in mean arterial pressure from baseline to follow-up time points. LETI: laryngoscopy and endotracheal intubation; T1 represents extubation time point; KeLiDex: ketamine, lignocaine, and dexmedetomidine

	KeLiDex (Group A)		Fentanyl (Group B)		
Timepoint Comparison	Absolute Change	P-value*	Absolute Change	P-value*	P-value^#^
Preoperative - Baseline	2.97 (15.61)	1.000	5.57 (10.00)	1.000	0.520
5 Minutes before LETI - Baseline	-2.10 (17.57)	1.000	-11.70 (21.84)	1.000	0.326
Post Intubation - Baseline	-3.20 (14.99)	1.000	-3.60 (21.47)	1.000	1.000
5 Minutes - Baseline	-0.10 (19.34)	1.000	-10.50 (25.07)	0.998	0.226
10 Minutes - Baseline	-10.00 (18.85)	1.000	-13.30 (24.74)	0.993	0.940
Incision - Baseline	0.20 (19.28)	1.000	-14.00 (22.26)	1.000	0.241
15 Minutes - Baseline	-13.10 (24.75)	0.976	-4.20 (19.24)	1.000	0.326
30 Minutes - Baseline	-15.00 (14.87)	0.796	-11.50 (16.28)	1.000	0.596
45 Minutes - Baseline	-19.40 (13.61)	0.590	-7.90 (17.00)	1.000	0.150
60 Minutes - Baseline	-18.60 (16.35)	0.656	-5.50 (17.56)	1.000	0.121
75 Minutes - Baseline	-22.20 (12.44)	0.200	-6.70 (18.52)	1.000	0.014
90 Minutes - Baseline	-23.90 (14.03)	0.106	-12.67 (17.41)	1.000	0.120
120 Minutes - Baseline	-19.90 (15.02)	0.403	-10.00 (16.54)	1.000	0.270
150 Minutes - Baseline	-19.83 (18.69)	1.000	-9.86 (14.76)	1.000	0.234
180 Minutes - Baseline	-17.75 (15.82)	1.000	-6.20 (19.75)	1.000	0.556
210 Minutes - Baseline	-28.50 (6.36)	1.000	-15.67 (17.90)	1.000	0.400
240 Minutes - Baseline	-27.50 (10.61)	1.000	-9.00 (13.75)	0.978	0.400
Reversal - Baseline	-9.30 (25.67)	1.000	-7.50 (24.46)	1.000	0.762
Extubation (T1) - Baseline	1.00 (23.84)	1.000	-3.60 (20.11)	1.000	0.970
1 Minute of T1 - Baseline	-3.10 (23.59)	1.000	-5.00 (20.61)	1.000	1.000
5 Minutes of T1 - Baseline	2.50 (24.51)	1.000	-7.20 (21.02)	1.000	0.449
10 Minutes of T1 - Baseline	-5.60 (27.56)	1.000	-3.70 (21.11)	1.000	0.791
20 Minutes of T1 - Baseline	-6.40 (24.11)	1.000	-4.60 (21.46)	1.000	0.853
30 Minutes of T1 - Baseline	-5.90 (24.11)	1.000	-4.80 (21.89)	1.000	0.734
40 Minutes of T1 - Baseline	-7.30 (23.40)	1.000	-6.60 (20.49)	1.000	0.705
50 Minutes of T1 - Baseline	-7.60 (22.42)	1.000	-8.80 (20.09)	1.000	1.000
60 Minutes of T1 - Baseline	-8.50 (19.28)	1.000	-7.70 (19.07)	1.000	0.820

**Figure 2:**
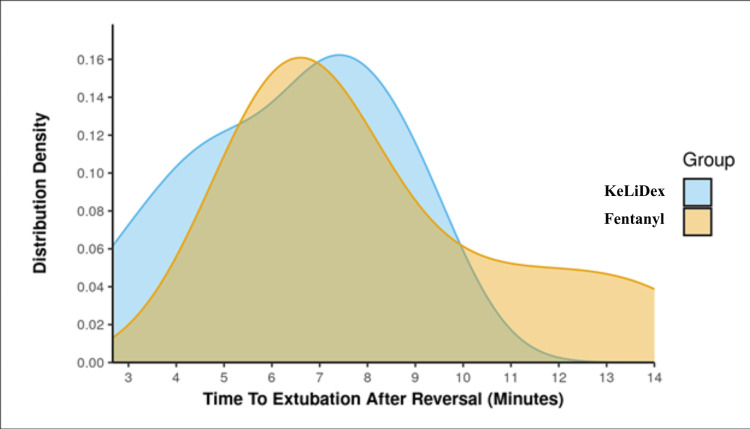
Distribution plot of the KeLiDex and fentanyl group for time to extubation after reversal. KeLiDex: ketamine, lignocaine, and dexmedetomidine

Both groups had similar anesthesia and surgical duration. The T-tEAR in Group A (KeLiDex) ranged from 2.67 to 9 minutes, while it ranged from 5 to 14 minutes in Group B (fentanyl). The mean ± SD of T-tEAR was 6.37 ± 2.13, and in Group B was 8.18 ± 2.92 minutes. The median (IQR) was 7 (5-7.75) and 7.25 (6.08-9.5), respectively, and the differences between the groups were statistically insignificant; W = 35.0, p = 0.27 with a strength of association (point-biserial correlation) = 0.35 (medium effect size). The distribution of T-tEAR is shown in Figure 2.

At 10 minutes of extubation, participants of both groups were slightly sedated with a mean RASS of -1. It improved to mean ± SD of -0.90 ± 0.32 and -0.80 ± 0.42 at 20 minutes in Group A (KeLiDex) and Group B (fentanyl), respectively. The score improved further to -0.20 ± 0.42 for both groups at 30 minutes. There was no statistical difference in the sedation score across the observation period. The mean Modified Alderette Score increased from a minimum of 8.60 at the 10-minute post-extubation point to a maximum of 10.00 at the 60-minute post-extubation point in Group A (KeLiDex). On the other hand, it increased from a minimum of 8.40 at the 10-minute post-extubation point to a maximum of 10.00 within the same time. While the intragroup changes were statistically significant for both groups (Friedman Test: χ2 = 44.6, p = <0.001), intergroup differences were insignificant at all the time points.

The NRS for pain at rest and movement in the KeLiDex group was slightly better than that of the fentanyl group. However, a statistically significant difference was not noted in NRS at any time point for NRS at rest and movement except for the 24-hour timepoint for NRS at movement (Table 3).

**Table 3:** Comparison of NRS presented as mean ± SD over different time points expressed as mean and standard deviation; p-value <0.05 was considered significant. NRS: numerical rating scale; SD: standard deviation * P-value for change in NRS over time within each group (Friedman Test). # Overall P-value for comparison of the change in NRS over time between the two groups (generalized estimating equations).

Time Points	NRS at Rest		P-value^*^	NRS at Movement		P-value^*^
	KeLiDex (Group A)	Fentanyl (Group B)		KeLiDex (Group A)	Fentanyl (Group B)	
10 Minutes Post Operative	3.7 ± 1.06	3.8 ± 1.48	0.548	3.9 ± 1.29	4.3 ± 1.83	0.391
30 Minutes Post Operative	3.9 ± 0.57	3.7 ± 1.42	0.824	4.0 ± 0.82	4.3 ± 1.70	0.234
60 Minutes Post Operative	3.7 ± 0.82	3.7 ± 1.42	0.578	3.9 ± 0.99	3.9 ± 1.52	0.803
120 Minutes Post Operative	3.4 ± 0.84	3.9 ± 0.57	0.125	3.7 ± 0.95	4.1 ± 0.57	0.316
6 Hours Post Operative	3.1 ± 0.57	3.5 ± 0.53	0.136	3.3 ± 0.95	4.0 ± 0.47	0.055
12 Hours Post Operative	3.1 ± 0.57	3.4 ± 0.52	0.259	3.2± 0.79	3.8 ± 0.42	0.062
24 Hours Post Operative	2.9 ± 0.74	3.1 ± 0.74	0.565	2.8 ± 0.79	3.6 ± 0.52	0.026
*P-value	<0.001	<0.001		<0.001	0.006	
^#^Overall P-value	0.475			0.203		

Although 80.0% of the participants in Group A required paracetamol only as a rescue analgesic, Fisher's exact test exploring the association between "Group" and "Rescue Analgesics" found no significant difference between the various groups regarding the distribution of rescue analgesics (χ2 = 2.892, p = 0.443).

The mean ± SD of time to rescue analgesics in Group A was 207.0 ± 222.58, while 70.50 ± 47.98 minutes in Group B. In terms of the median (IQR), the values were 142.5 (75-180) and 67.5 (32.5-120) minutes for Group A and Group B, respectively. The groups had no significant difference; W = 75.500, p = 0.055. The PONV incidence was also statistically indifferent among the group; p-value 0.473.

## Discussion

The present pilot study showed that KeLiDex used as an alternative to fentanyl (opioid), was equally effective and probably even better in maintaining intraoperative hemodynamic stability under controlled balanced anesthesia. However, all the drugs used for intervention had a short duration of action, and the residual effects did not last in any group for a prolonged duration. The QLB-2 was performed in both groups; the doses used in both groups were similar, and 15 mL remained within the safe limit for even the KeLiDex group. The analgesia technique, however, was ineffective as the sole strategy. It required rescue analgesia to keep the NRS <4/10. Nonetheless, the requirement mainly was for paracetamol, reiterating the value of such blocks as part of multimodal analgesia, opioid-sparing analgesia, and OFA. During the observation period, the sedation scores were within acceptable limits of -1 to 0 (zero), and no statistically significant differences were noted between the groups. The overall change in RASS over time in the two groups, as compared using the generalized estimating equations method, also showed indifference in the trend of sedation scores. Similar results were also found for recovery profile and PACU discharge readiness, indicating that KeLiDex can be an acceptable alternative to traditional opioid (fentanyl) based anesthesia.

Perioperative use of dexmedetomidine and lignocaine for analgesia and as an opioid-sparing or replacement agent for OFA is well described in the literature [11-13]. However, lignocaine needs a higher dose as the sole analgesic and leads to sedation [14]. Further, its hemodynamic attenuation to laryngoscopy, ETI, and the intraoperative period is also well known [15]. However, the results for postoperative opioid-sparing are not consistent [16]. Although ketamine is a potent analgesic, it has significant side effects and even has addiction potential. Therefore, we planned to evaluate low dosages of these drugs in combination (KeLiDex) as an opioid alternative for OFA. The present study could not find a significant difference in the postoperative sedation level among the groups throughout the observed period. No oversedation was noted in all patients, which might be explained by the minimal effective dosage of all intervention drugs. Further, dexmedetomidine causes conscious sedation, which is also unlikely to affect the modified Aldrete score used for assessing the readiness for discharge from PACU.

A few studies have examined the drugs in different combinations in the non-opioid category and were found effective in the management of pain and outcome of the patient [17-20]. Luong used lignocaine infusion and ketamine and ketorolac bolus for laparoscopic cholecystectomy as an OFA method [17]. The study found that the depth of anesthesia, response, hemodynamics, and pain were comparable to opioid-based anesthesia [17]. In a study, Vishnuraj et al. used ketamine and dexmedetomidine for OFA and found them effective, and the mixture also reduced the analgesic requirement for two hours postoperatively [18]. In another case series, a dexmedetomidine, ketamine, and lignocaine combination was used as an OFA modality for laparoscopic cholecystectomy [19]. A nearly similar drug combination, i.e., dexmedetomidine-esketamine-lidocaine, has also been used as opioid-free total intravenous anesthesia for lumpectomy by Qian et al. [20]. The study enrolled 80 females and used sufentanil and remifentanil for opioid-based anesthesia, whereas we used fentanyl. Further, the study used esketamine, but we used ketamine. Nevertheless, the results of the present study and the study by Qian et al. for the pain scores and hemodynamics variations were similar [20]. Lignocaine, paracetamol, and magnesium sulphate combinations in various dosages with or without tramadol have been described as a part of the Shiv mix [21]. It has been used as a multimodal analgesia and to prevent hemodynamic instability during laryngoscopy and endotracheal intubation (LETI) and the intraoperative period for various surgeries [21,22]. While the combination without tramadol can be considered OFA, the efficacy of such a mix needs to be further evaluated in well-designed studies.

In our study, we did not find any PONV in the fentanyl group, and only two cases of PONV scored on the Likert Scale of 1 and 2 in the KeLiDex group; the difference was statistically insignificant. A systematic review and meta-analysis findings show that even opioid-based anesthesia did not ensure a pain-free postoperative period; instead, it led to increased PONV, while OFA had the advantage of improved postoperative outcomes, indicating that OFA can effectively be practiced for a broader range of patients [23,24]. Although statistically insignificant, the present study findings of low absolute numbers of PONV in the opioid-based anesthesia group contradict the existing literature and evidence [23,24]. This might be explained by using prophylactic ondansetron and a smaller sample. Further, the groups might have an unequal distribution of high-risk patients.

Even though the differences were statistically insignificant, it has practical applications. The indifference of hemodynamic variation, pain scores, rescue analgesic requirements, the sedation caused, and time to recovery between KeLiDex and fentanyl indicates that KeLiDex can replace fentanyl-based anesthesia and promote OFA in the present era of the opioid crisis. Despite using multiple drugs (KeLiDex), the T-tEAR was similar to fentanyl-based anesthesia, which also indicates that the KeLiDex can be safely used without a fear of delayed recovery.

The study has a few limitations that also need to be mentioned. It is a pilot study conducted in a single center. As pilot studies usually require smaller samples, the sample size included might not truly represent a well-powered study, as seen for the PONV results in our study. Although we did randomization and tried to maintain concealment using unique codes, followed strict inclusion and exclusion criteria, and enrolled consecutive eligible patients to mitigate biases, blinding was feasible only for the participants. Economic and operational constraints also need to be considered. The drugs were diluted in a 50 mL syringe and given via infusion pumps. Multiple infusion pumps were needed for the patients in the intraoperative period, requiring extra intravenous lines, especially in the KeLiDex group. KeLiDex infusion was stopped immediately after the removal of the specimen, and our findings might not apply to the procedure when we want to continue the injection further into the postoperative period. Further, we still considered opioids (tramadol) as a rescue in the postoperative period, whereas the OFA horizon should also include the postoperative period. Therefore, there is a need for a well-powered study to have a better insight, and the present study can serve as a basis for such future studies.

## Conclusions

KeLiDex administered in analgesic doses to the patients posted for laparoscopic nephrectomies could effectively control intraoperative hemodynamics well in line with the opioid-based anesthesia. Further, recovery from the anesthesia, sedation, and PACU discharge readiness was also like that of fentanyl (opioid)-based anesthesia. The postoperative pain, both at rest and in movements, was also comparable. Although the present findings prove our hypothesis that the KeLiDex-based OFA can be similarly effective or even better than fentanyl-based anesthesia, the present study is a pilot one, and future well-powered studies will be required for better insight. Further, we also need to evaluate the financial aspect of KeLiDex use in terms of equipment required and drug cost, especially for developing countries where the per capita health expenditure and purchasing power are limited.
